# Foreign Bodies in the Conjunctival Sac

**Published:** 1892-03-05

**Authors:** 


### OPHTHALMOLOGY.

### FOREIGN BODIES IN THE CONJUNCTIVAL SAC.

Foreign bodies entering the conjunctival sac are among the commonest causes of every-day injury to the eye. The fly which frequents lime trees seems especially fond of finding a resting place within the human eyelid; other insects occasionally find their way there, but more common still are specks of dust, coal dust, iron rust, and such bodies. Fragments of iron, steel, brass, stone, in fact, any metals or minerals being ground, cut, or broken, are liable to find their way into the eyes of the manipulators or bystanders. Chaff and seeds are wafted thither by the fan or the wind; eyelashes find their way in, instead of falling outwards. All these various substances cause much annoyance and disturbance of the surrounding part, and produce inflammations of the conjunctiva and cornea, and if not discovered and removed, may go on to serious results, ending in sympathetic inflammation in the other eye.

The patient generally comes with a pretty clear history, stating that something has gone into his eye, and that he can feel it when he moves his lid, or else that inflammation of the eye has come on quite suddenly, with much pain and lachrymation. When the surgeon gets such a history he should examine for a foreign body. In a large proportion of cases he will find the offending body lying on the inner or conjunctival surface of the upper eyelid. To evert the eyelid, fix and depress the lid with the forefinger applied to the anterior surface of the lid, and with the thumb of the same hand roll the edge of the eyelid upwards and forwards; or the handle of a knife or probe being laid across the base of the cartilage of the lid, with the forefinger and thumb of the hand not holding the knife seize the lid, pull it slightly downwards, and turn it up over the knife-handle, penholder, probe, or whatever similar instrument is used.

If the foreign body is not found on the conjunctival surface of the upper eyelid, examine the corresponding surface of the lower lid by drawing it down with the forefinger, and carefully examine the "cul de sacs" where the palpebral and ocular conjunctiva join, as in some persons they are deep, and may easily conceal a large body. Thus we have known a seed of barley remain concealed there for nearly three weeks, and when found, to be actually sprouting. During this interval the eye was examined, and history relates the eyelids to have been everted by several unskilled persons. If not found in these situations search the corneal surface. Black particles of coal dust, &c., not imbedded in the tissue, may be easily detected by the unaided eye, but for small bodies and bright metals the oblique light is generally necessary with or without a magnifying lens. Oblique illumination consists in concentrating the rays of light from a lamp on to the object to be examined by means of a convex lens. The lens ordinarily used in examining by the indirect ophthalmoscopic method is suitable—*i.e.*, +13D. The light is placed opposite to, or slightly to one side, of the patient at about two feet distance, and the lens held so that the rays are focussed on the cornea, and by this means the smallest speck of metal is discoverable.

The removal of foreign bodies from the superficial parts of the eye is most readily accomplished by means of the corneal spud, a short, narrow steel instrument, blunt along the sides of the blade, but sharper at the point, which is about 1·24th in. broad, flattened on one surface, and convex on the other. There is another form equally, if not more useful, in which the one surface is grooved instead of being flat. Armed with one of these, the eye having had a few drops of a 4 per cent. solution of cocain in water instilled into it, any foreign body can be removed, those imbedded in the corneal tissue are the most difficult to get rid of, and require often some time and skill, as no injury must be done to the surrounding cornea, beyond disturbing its epithelial layer. After the offending material has been removed, no further treatment is needed, unless it has been imbedded in, or scratched the cornea, when cocain in castor oil should be applied, which diminishes the friction of the lid against the wounded surface; and in bad cases the eyelid should be stopped from blinking by applying a pad and bandage to its outer side when closed. For removing flies or fragments from the inner surface of the eyelids, or from the cornea when not embedded, the corner of a handkerchief rolled up is the best and simplest instrument to use, as the metallic spud described above, in an unaccustomed hand, is liable to cause slight abrasions. The workmen in our factories are skilled at everting the eyelid and removing foreign bodies with the handkerchief, so in hospital practice it is as a rule only the more complicated cases that we see, where the fragment is imbedded, or has only been partially removed.

